# Antimicrobial Activity of Mast Cells: Role and Relevance of Extracellular DNA Traps

**DOI:** 10.3389/fimmu.2016.00265

**Published:** 2016-07-18

**Authors:** Helene Möllerherm, Maren von Köckritz-Blickwede, Katja Branitzki-Heinemann

**Affiliations:** ^1^Department of Physiological Chemistry, University for Veterinary Medicine Hannover, Hanover, Germany; ^2^Research Center for Emerging Infections and Zoonoses (RIZ), University for Veterinary Medicine Hannover, Hanover, Germany

**Keywords:** MCET, extracellular traps, mast cell, neutrophil, innate immunity, antimicrobial activity, phagocytosis, degranulation

## Abstract

Mast cells (MCs) have been shown to release their nuclear DNA and subsequently form mast cell extracellular traps (MCETs) comparable to neutrophil extracellular traps, which are able to entrap and kill various microbes. The formation of extracellular traps is associated with the disruption of the nuclear membrane, which leads to mixing of nuclear compounds with granule components and causes the death of the cell, a process called ETosis. The question arises why do MCs release MCETs although they are very well known as multifunctional long-living sentinel cells? MCs are known to play a role during allergic reactions and certain parasitic infections. Nonetheless, they are also critical components of the early host innate immune response to bacterial and fungal pathogens: MCs contribute to the initiation of the early immune response by recruiting effector cells including neutrophils and macrophages by locally releasing inflammatory mediators, such as TNF-α. Moreover, various studies demonstrate that MCs are able to eliminate microbes through intracellular as well as extracellular antimicrobial mechanisms, including MCET formation similar to that of professional phagocytes. Recent literature leads to the suggestion that MCET formation is not the result of a passive release of DNA and granule proteins during cellular disintegration, but rather an active and controlled process in response to specific stimulation, which contributes to the innate host defense. This review will discuss the different known aspects of the antimicrobial activities of MCs with a special focus on MCETs, and their role and relevance during infection and inflammation.

## Introduction

Mast cells (MCs) have become famous for their role in type I hypersensitivity reactions. Better known as IgE-mediated allergic reactions, this MC response is induced after multivalent cross-linkage of antigens with antigen-specific IgE, which then bind to high-affinity IgE receptors (Fc∈RI) on the cellular surface (1–3). For a long time, MCs have been underestimated and mainly known for their role as mediators in the early and acute phases of allergic reactions as well as their activation during certain parasitic infections (4). Indeed, they hold a multitude of very important functions in the innate and adaptive host immune responses against bacterial and fungal pathogens (5, 6) (see Table 1).

**Table 1:** **Interaction of MCs with selected pathogens**.

Pathogen	Mast cell type	Phagocytosis	MCETs	Degranulation	Reference
*Staphylococcus aureus*	CBHMC	√ no		√	(7, 10)
	HMC-1		√		(8, 9)
	BMMCs		√		(8)
*Streptococcus pyogenes*	BMMC	no	√	√	(9)
	HMC-1	no	√		(9,11)
*Pseudomonas aeruginosa*	Murine skin mast cells			√	(12)
	HMC-1		√		(9)
*Citrobacter freundii*	CBHMC	√			(7)
*Klebsiella pneumoniae*	CBHMC	√			(7)
	Mouse lung mast cells *in vivo*			√	(13)
*Escherichia coli*	CBHMC	√			(7)
	Mouse lung mast cells *in vivo*			√	(13,14)
*Streptococcus faecium*	CBHMC	√			(7)
*Citrobacter rodentium*	BMMC			AMP	(14)
*Enterococcus faecalis*	BMMC		√	√	(15)
*Candida albicans*	HMC-1, CBHMC		√	√	(16)
*Listeria monocytogenes*	BMMC	no		√	(17)
*Salmonella typhimurium*	BMMC	no		√	(17)
*Trichinella spiralis*	RBL-2H3, BMMC			√	(18)
*Leishmania major*	*in vivo*			√	(19)
*Helicobacter pylori*	BMMC, RBL-2H3 cells			√	(20)

MCs derive from hematopoietic progenitor cells and circulate in the blood until they reach their destination in the tissues, where they differentiate under the influence of growth factors and cytokines that ultimately determine their mature, long-living phenotype (3). Different subsets of mature MCs have been described on the basis of functional, structural, and biochemical characteristics. Consequently, they are classified into at least two subgroups: mucosal MCs (MMCs) and connective tissue-type MCs (CTMCs) (3, 21). CTMCs typically reside in the skin and the peritoneal cavity. In contrast, MMCs are predominant in the mucosal layer of the intestine, where their numbers expand dramatically during e.g. parasitic infections (22, 23). Considering their long life span and phenotypic plasticity in the tissues, MCs contribution in chronic or acute infections is not fully understood (24). They are largely distributed near interfaces and potential entry sites of pathogens, such as the skin, the respiratory, and intestinal mucosa, and in close proximity to blood vessels and nerve cells (25, 26); therefore, MCs belong to the first immune cells, which come in contact with intruders. Since they orchestrate the immune response by releasing various mediators, these long-living sentinel cells are crucial for the early recruitment of effector cells (24).

The main function of MCs is the release of inflammatory mediators such as proteases, cytokines, and chemokines by degranulation into the surrounding environment (22, 27). MCs are the only cell type known to pre-store TNF-α in their secretory granules, which can be released immediately upon activation e.g., by pathogens to initiate the early phase of the inflammatory response (13, 28). Rocha-de-Souza et al. (10) showed that both alive and dead *Staphylococcus (S.) aureus* trigger TNF-α and IL-8 release from cord-blood-derived MCs in a time-dependent manner. Nakamura et al. (29) published that culture supernatants of *S. aureus* contain potent MC degranulation activators. Biochemical analysis identified δ-toxin as the MC degranulation-inducing factor produced by *S. aureus* (30). Importantly, skin colonization with *S. aureus*, but not a mutant deficient in δ-toxin, promoted IgE and IL-4 production as well as skin diseases. Dietrich and colleagues showed that, in response to toll-like receptor (TLR) activation by the Gram-positive and -negative bacteria *Listeria (L.) monocytogenes* and *Salmonella (S.) typhimurium*, respectively, MCs elicit proinflammatory, but not type I IFN responses. In contrast, the response to viral infection is type I IFN dependent. Type I IFN signaling attenuates mast cell-dependent neutrophil recruitment that is required for bacterial clearance. Thus, the fact that MCs are equipped with the ability to release type I IFNs, but mount proinflammatory responses only upon TLR activation by bacteria, illustrates how MCs adjust their responses for optimal antibacterial and antiviral host defenses (17).

MCs are highly efficient effector cells that do not only release inflammatory mediators but also different antimicrobial peptides (AMPs), such as cathelicidins (31). These peptides have cationic and amphipathic properties that promote interactions with biological membranes and selectively kill a wide spectrum of microbes including bacteria, fungi, enveloped viruses, and protozoa (31). *In vivo* evidence from MC- and cathelicidin-deficient mouse models indicates that MC cathelicidins modulate tissue responsiveness to bacterial infection (32). The authors suggested that cathelicidins act as a natural antibiotic in MCs and may protect the skin from invasive group A *Streptococcus* (GAS) and *S. aureus* infection by direct bacterial killing. Moreover, the presence of cathelicidin in MCs may act to facilitate recruitment of neutrophils, thus indirectly providing enhanced protection against infection.

Despite the fact that the MCs release key inflammatory compounds to modulate the immune response and to fight pathogens with AMPs, the cells are additionally discussed to be able to eliminate bacteria through an intracellular bactericidal mechanism similar to that of professional phagocytes (33). This mechanism seems to involve the endosome–lysosome pathway, in which the bacteria are killed through a combination of oxidative and non-oxidative killing systems (33). Arock and coworkers showed that human cord blood MCs (CBHMCs) are able to phagocytose and kill *S. aureus, Streptococcus faecium, Klebsiella pneumoniae, Citrobacter freundii*, and *Escherichia (E.) coli* by scanning and transmission electron microscopy and by quantifying bacterial survival in the presence of MCs compared with human umbilical vein endothelial cells (HUVECs) (7). However, although the authors compared *in vitro* results generated by different techniques with distinct pathogens, the data still remain short of a definitive proof in respect to the phagocytic response, since only total killing of bacteria in the presence of MCs was measured, and not a specific intracellular killing as normally performed by a gentamicin-protection assay. Finally, in contrast to the above hypothesis that MCs are able to phagocytose, Dietrich et al. found only an exclusive extracellular interaction of MCs with *L. monocytogenes* or *S. typhimurium* using transmission electron microscopy (17).

Interestingly, Abel et al. (34) showed that MCs can internalize *S. aureus* without subsequent intracellular killing: the *S. aureus* strain SH100 was shown to be internalized *in vitro* by murine and human MCs (primary murine bone marrow-derived MCs “BMMCs,” and human carcinoma MC line “HMC-1”) and by skin MCs during *in vivo* infections. MCs are utilized as a vehicle and a safe intracellular niche providing protection against other immune cells. Since the internalization efficiency depends on bacterial viability, the authors hypothesized that *S. aureus* may actively induce its uptake into MCs (34). In a recent follow-up publication, Goldmann and colleagues demonstrated enhanced *S. aureus* internalization by MCs based on the interaction of staphylococcal fibronectin-binding protein with host cellular integrin β1 (35). However, aside from the fact that *S. aureus* mediates its own uptake into MCs to evade immune cell killing, MCs have been shown by several authors to exert a direct antimicrobial activity against this and other pathogens (7, 9, 34). Thus, it may be assumed that although MCs may act as a long-term staphylococcal reservoir supporting persistence and chronic carriage, their activation can help to limit the early pathogen burden in the infected host.

In summary, MCs do not only orchestrate the early innate immune response through the release of mediators but they can also act antimicrobially in a pathogen-specific manner: extracellular by the release of antimicrobial products such as the cathelicidin-related AMPs (36) or intracellular by a phagocytic process. Finally, in 2008, an additional antimicrobial strategy was described for MCs, which was already known for neutrophils: the formation of antimicrobial mast cell extracellular traps (MCETs) (9).

## Mast Cell Extracellular Traps

Similar to the formation of neutrophil extracellular traps (NETs), MCs have also been shown to release their nuclear DNA and subsequently form antimicrobial MCETs that resemble extracellular dendritic extensions (9). Interestingly, already in 1989, Trotter and colleagues mentioned that “superficial MCs have a smaller size and may be dendritic, with relatively few granules […]” (37). However, the first experimental study on MCs and the formation of antimicrobial extracellular structures that strongly resembled the recently described NETs was published by von Köckritz-Blickwede et al. (9). The authors showed that even though MCs are unable to phagocytose *Streptococcus (S.) pyogenes*, they still can efficiently inhibit the growth by the release of MCETs. MCETs were found to support the extracellular killing of clumped bacteria that were not efficiently phagocytosed. Those MCET fibers are composed of DNA, histones, the MCs-specific protease tryptase, and AMPs such as the cathelicidin AMP LL-37 (9).

Detailed information about the specific trigger that initiates MCET formation and the mechanisms regulating the removal of MCETs is still missing. However, literature clearly demonstrates that MCET formation is not the result of a passive release of DNA and granule proteins during cellular disintegration but, rather, an active and controlled process similar to that described for NETs (9). Similar to the observations of Fuchs et al., who has implicated the production of reactive oxygen radicals and induction of cell death in the production of NETs, the formation of MCETs also strongly depends on the production of reactive oxygen species (ROS) and results in the death of the MC. Examination of stimulated MCs by electron and fluorescent microscopy confirmed that MCs undergo a similar mechanism of cell death as described for neutrophils when releasing NETs: MCET formation is associated with the disruption of the nuclear membrane before nuclear and granular components mix causing the death of the cell. Although ROS had been previously associated with the induction of neutrophil apoptosis, Fuchs et al. showed that the process accounting for NET formation is neither typical apoptosis nor necrosis, but rather a new form of ROS-dependent cell death recently termed “NETosis” (38). Since this is also true for the formation of MCETs, this cell death was also named “ETosis” (39). The formation of MCETs can be greatly increased after stimulation of MCs with phorbol-12-myristate-13-acetate (PMA), similar to NETs (40) or with the H_2_O_2_-producing enzyme glucose oxidase, which is another indicator for the ROS-dependent MCET formation (9). Nevertheless, a key question still needs to be answered: why do MCs release MCETs although they are very well known as multifunctional long-living sentinel cells – what is worth dying for?

Several publications show that the formation of MCETs represents a novel mechanism by which MCs contribute to host defenses against bacterial and fungal pathogens. Interestingly, a diffused gradient of extracellular tryptase staining was often observed in areas with large numbers of bacteria during *in vivo* infections, which may indicate a massive release of this enzyme and possibly the not clearly visible but occurring formation of MCETs at the site of infection. The first specific bacterial protein identified to promote MCET production was the *S. pyogenes* surface M1 protein (11). In quantitative assays, loss of M1 protein in the *S. pyogenes* ΔM1 mutant resulted in a significant decrease in the stimulation of NET as well as MCET release. Despite its role in inducing ET formation, the authors found M1 protein promoting extracellular bacterial survival, at least in part due to resistance to the human AMP LL-37, an important effector of bacterial killing associated with extracellular traps. LL-37 and its murine analog CRAMP are stored in MC granules; its expression is upregulated by LPS and found within the MCET structures where it contributes to the antimicrobial activity (9, 31). It has already been shown for neutrophils that LL-37 significantly facilitated NET formation by primary human blood-derived neutrophils alone, in the presence of the classical chemical NET inducer PMA or in the presence of *S. aureus* (41). Nonetheless, the role of LL-37 in MCETs still needs to be investigated. Interestingly, Scheb-Wetzel et al. recently showed that MC release extracellular traps in response to *Enterococcus (E.) faecalis*, and the sensitivity of this pathogen to the antimicrobial effect of cathelicidin LL-37 indicated a potential major role for this AMP in the antimicrobial activity of MCs against *E. faecalis* (15). However, the level of MCET formation was not as pronounced as it has been reported for other pathogens such as *S. pyogenes* (9); therefore, killing of *E. faecalis* by MCETs cannot fully account for the antimicrobial effect of MCs observed in this study. This was further confirmed by the diminished, but still significant, antimicrobial effect of MCs after dismantling of the MCETs by nuclease treatment. The reason why only a certain percentage/population of MCs in the cultures released MCETs is not yet clear; eventually, this phenomenon may reflect heterogeneity in the physiological status of the MCs in culture; a similar phenomenon has been reported for extracellular trap formation by neutrophils (38).

Importantly, Lopes et al. (16) showed that MCs reveal an antimicrobial activity against higher eukaryotes, namely the fungi *Candida (C.) albicans*. MCET release appears to be a mechanism of immune defense present in the MC toolbox against fungal pathogens, as both primary cells and HMC-1 release MCETs upon *C. albicans* stimulation. Interestingly, in contrast to bacteria (8, 9, 11, 15), *C. albicans* viability was not affected by the MC-derived DNA fibers and thus MCETs rather contribute to physical restriction of this fungal pathogens. Finally, the exact mechanism and the *in vivo* relevance of *C. albicans*-induced MC death need to be determined in further studies.

Similar to what has been shown for NETs, the formation of MCETs also seems to be associated with detrimental effects during health and diseases: a novel mechanistic stimulus for the release of extracellular traps in psoriasis lesions was described by Lin et al. (42). It was demonstrated that IL-23 and IL-1β increased the numbers of extracellular trap forming cells e.g., neutrophils and MCs contributing to the release of the pathogenic cytokine IL-17. Nevertheless, the precise signaling mechanisms regulating this process remain to be defined. These observations support a model in which MCs and neutrophils play significant roles in the pathophysiology of psoriasis and potentially other autoinflammatory diseases driven by the IL-23-IL-17 axis. The authors suggested that a modulation of MC and neutrophil ETosis and release of IL-17 could be used as a novel therapeutic mechanism of action for drugs targeting IL-23.

## Regulation of the Formation of MCETs: Comparison with NETs

It is still not entirely clear how the formation of MCETs is transcriptionally regulated. One factor identified to contribute to formation of MCETs is the central transcriptional regulator of the cellular response to hypoxic stress, namely the hypoxia-inducible factor 1α (HIF-1α). Oxygen stress or hypoxia occurs in tissues during an infection, mostly, due to overconsumption of oxygen by pathogens and recruited immune cells. Importantly, HIF-1α activation or stabilization has been shown to support myeloid cell production of defense factors and improved bactericidal capacity of immune cells (43, 44). In good correlation, Branitzki-Heinemann et al. (8) showed that MCET release was enhanced after MCs were treated with AKB-4924, a HIF-1α stabilizing agent. These MCETs were able to entrap and immobilize *S. aureus*. Inhibition of phagocytosis did not alter the antimicrobial activity of MCs, whether or not HIF-1α activity was boosted with AKB-4924. Augmentation of HIF-1α-activity resulted in a boosting of the antimicrobial activity of human and murine MCs by inducing MCET formation. The results show for the first time that the extracellular antimicrobial activity of MCs is transcriptionally regulated and support the assumption that the transcription factor HIF-1α is not only a global player in the cellular response to low oxygen stress but also may, furthermore, act as a key regulator of the antimicrobial response of several immune cells including MCs (43–47). However, in contrast to MCs, the role of HIF-1α in the formation of extracellular traps produced by neutrophils remains to be elucidated. It has been shown that some well-known HIF-1α agonists including mimosine and desferrioxamine (DFO) have an impact in neutrophil function. Mimosine has been proven to boost the bacterial killing by neutrophils. This effect was eliminated after DNAse treatment suggesting the involvement of NETs in the mimosine-mediated neutrophil killing activity (48). In good correlation to these data, DFO was recently described as a positive stimulus for NET formation (49). The authors hypothesized that stabilization of HIF-1α with agonists, such as DFO or mimosine, might facilitate the formation of NETs, which confirmed results from McInturff and coworkers showing that HIF-1α contributes to rapamycin-induced NET formation in human neutrophils (50).

Although, MCETs and NETs share common characteristics, there are several cell type-specific differences. The formation of MCETs can be greatly increased after stimulation of MCs with PMA prior to infection (9), similar to NETs (40). But interestingly, MCs release less MCETs stimulated with the same stimuli in comparison to neutrophils when studying the respective literature: whereas more than 90% of neutrophils undergo ETosis within 3 h upon stimulation with PMA (38), approximately only 40% of MCs undergo ETosis after 6 h of stimulation with the same stimulus and concentration (9). Another important difference between MCs and neutrophils are their components, which are embedded in the DNA structures: elastase and MPO are essentially involved in the formation of NETs by degradation of histones and subsequent decondensation of chromatin; later both enzymes perform an antimicrobial role in NETs (51, 52). In MCs, elastase and myeloperoxidase are not even expressed (53). Until now, it is unclear if, for example, MC-specific tryptase, which has also been shown as a component of MCETs (9), has similar functions in MCs.

**Figure 1:**
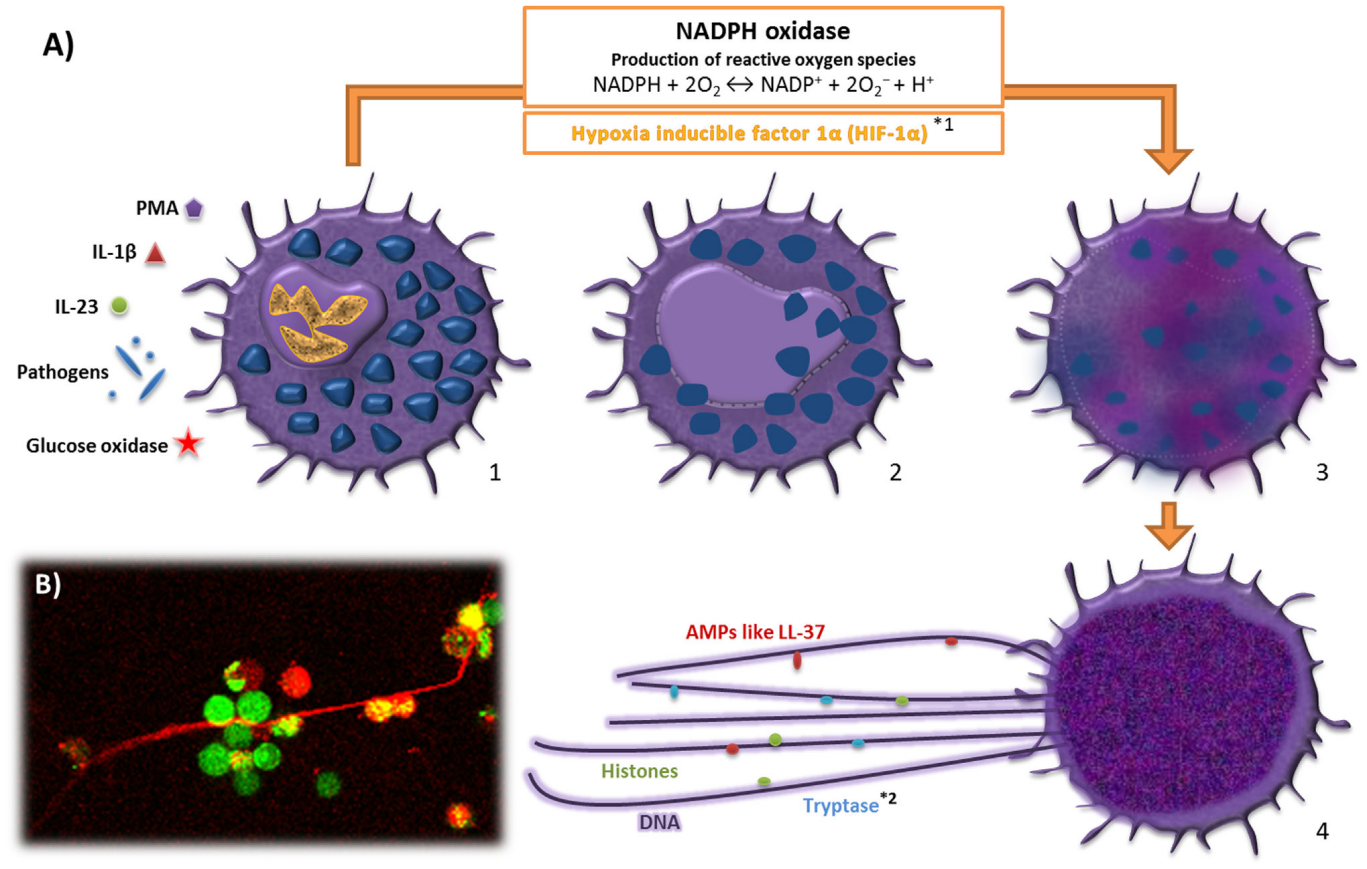
**Model for the formation of mast cell extracellular traps**. **(A)** 1. MCs are activated by contact with microbial pathogens different stimuli such as IL-1β, IL-23, PMA, and glucose oxidase. Stimulation of MCs results in the activation of NADPH oxidases and the formation of reactive oxygen species (ROS). 2. The nuclear membrane disrupts and the chromatin decondensates. 3. The nuclear contents mix with cytoplasmic and granular proteins. 4. Nuclear and granular components are released by the cell generating extracellular traps, which have the ability to entrap and/or kill different microbes, while also enhancing proinflammatory innate immune responses. **(B)** PMA-stimulated MCETs of BMMCs [live dead staining, red: dead cells (loss of integrity of the plasma membrane) and extracellular DNA, green: living cells (intracellular esterase activity)]. *Differences compared with neutrophil extracellular traps (NETs); ^1^Role of HlF-lα in the formation of NETs in contrast to MCETs is not yet exactly clarified and *^2^Tryptase is unique for MCETs.

In neutrophils, it has been additionally demonstrated that these cells can release ETs in response to infection, while remaining in a viable status, confirmed *in vivo* in a murine *S. aureus* skin infection model (54, 55). Formation of extracellular traps by viable eosinophils and basophils was also shown in response to *E. coli* and *S. aureus* (56, 57) and subsequent release of mitochondrial DNA (54). However, if a similar phenomenon also occurs in MCs is still unknown. Interestingly, in Figure 1, we can identify viable MCs in close contact to MCETs after treatment of MCs with PMA for 3 h. Thus, further investigations are needed to determine if MCs are also able to release extracellular traps in a viable status.

## Conclusion

In summary, the actual literature assumes that MCET formation is not the result of passive release of DNA and granular proteins during cellular disintegration, but rather an active and controlled process in response to specific stimulation. The extracellular structures act antimicrobially through a combination of direct killing of the entrapped pathogen or by its physical immobilization that enables the recruited effector cells to eliminate the pathogen. Both aspects may have a significant impact on the disease outcome. The fact that aggregated NETs limit inflammation by degrading cytokines and chemokines has already been shown (58). If MCET formation may decrease uncontrolled mast cell degranulation and subsequent dissemination of chemokines and cytokines during an acute overwhelming infection or autoimmune disease, which could lead to tissue damage, inflammation, and nerve cell activation with a potentially negative impact on the organism needs further investigation (59, 60). In any case, a plausible answer to the question “why do these long-living cells form MCETs” could be: MCETs are of particular importance in the direct and indirect antimicrobial activity against various pathogens; additionally, MCET formation may help to avoid or minimize affliction of the host by restricting the inflammatory responses.

## Author Contributions

All authors listed have made substantial, direct, and intellectual contribution to the work and approved it for publication.

## Conflict of Interest Statement

The authors declare that the research was conducted in the absence of any commercial or financial relationships that could be construed as a potential conflict of interest.
